# Hyperphosphatemia with elevated serum PTH and FGF23, reduced 1,25(OH)_2_D and normal FGF7 concentrations characterize patients with CKD

**DOI:** 10.1186/s12882-021-02311-3

**Published:** 2021-03-30

**Authors:** Kittrawee Kritmetapak, Louis Losbanos, Taylor E. Berent, Susan L. Ashrafzadeh-Kian, Alicia Algeciras-Schimnich, Jolaine M. Hines, Ravinder J. Singh, Rajiv Kumar

**Affiliations:** 1 Division of Nephrology and Hypertension, Department of Internal Medicine, Mayo Clinic, 200 1st Street SW, MN 55905 Rochester, USA; 2 Division of Nephrology, Department of Medicine, Faculty of Medicine, Khon Kaen University, Khon Kaen, Thailand; 3 Clinical Immunoassay Laboratory, Mayo Clinic, MN Rochester, USA; 4 Department of Laboratory Medicine and Pathology, Mayo Clinic, MN Rochester, USA; 5 Immunochemical Core Laboratory, Mayo Clinic, MN Rochester, USA; 6 Department of Biochemistry and Molecular Biology, Mayo Clinic, MN Rochester, USA

**Keywords:** Chronic kidney disease, Fibroblast growth factor, Parathyroid hormone, Phosphate, Vitamin D

## Abstract

**Background:**

Hyperphosphatemia confers adverse cardiovascular outcomes, and commonly occurs in late-stage CKD. Fibroblast growth factor 7 (FGF7) is a phosphaturic peptide which decreases renal phosphate transport *in vitro* and *in vivo*. Serum FGF7 concentrations are reduced in hyperphosphatemic patients with hypophosphatasia and are elevated in some hypophosphatemic patients with tumor-induced osteomalacia. No data, however, are available on whether circulating FGF7 concentrations increase to compensate for phosphate retention in CKD patients.

**Methods:**

This was a cross-sectional study performed among 85 adult patients with varying estimated glomerular filtration rates (eGFR). We measured serum intact FGF7 (iFGF7) concentration using an iFGF7 immunoassay and determined its associated factors. Relationships between eGFR and mineral metabolism biomarkers [phosphate, iFGF7, iFGF23, parathyroid hormone (PTH), and 1,25-dihydroxyvitamin D (1,25(OH)_2_D)] were explored.

**Results:**

For eGFRs of ≥ 60 (*n* = 31), 45–59 (*n* = 16), 30–44 (*n* = 11), 15–29 (*n* = 15), and < 15 mL/min/1.73 m^2^ (*n* = 12), median (IQ25-75) iFGF7 concentrations were 46.1 (39.2–56.9), 43.1 (39.0-51.5), 47.3 (38.3–66.5), 47.7 (37.7–55.8), and 49.6 (42.5–65.6) pg/mL, respectively (*P* = 0.62). Significant increases in serum iFGF23, PTH, and phosphate were observed at eGFRs of < 33 (95 % CI, 26.40-40.05), < 29 (95 % CI, 22.51–35.36), and < 22 mL/min/1.73 m^2^ (95 % CI, 19.25–25.51), respectively, while significant decreases in serum 1,25(OH)_2_D were observed at an eGFR of < 52 mL/min/1.73 m^2^ (95 % CI, 42.57–61.43). No significant correlation was found between serum iFGF7 and phosphate, iFGF23, PTH or 1,25(OH)_2_D. In multivariable analyses, body mass index (per 5 kg/m^2^ increase) was independently associated with the highest quartile of serum iFGF7 concentration (OR, 1.20; 95 % CI, 1.12–1.55).

**Conclusions:**

Compensatory decreases in circulating 1,25(OH)_2_D and increases in circulating iFGF23 and PTH, but not iFGF7, facilitate normalization of serum phosphate concentration in early stages of CKD. Whether other circulating phosphaturic peptides change in response to phosphate retention in CKD patients deserves further study.

## Background

Chronic kidney disease-mineral and bone disorder (CKD-MBD) is characterized by deranged metabolism of calcium, phosphate, parathyroid hormone (PTH), fibroblast growth factor 23 (FGF23), and 1,25-dihydroxyvitamin D (1,25(OH)_2_D).[1–3] In addition to changes in calciotropic and phosphotropic factors, CKD-MBD is frequently associated with bone abnormalities and vascular calcifications, which contribute to the substantial burden of cardiovascular disease in patients with CKD.[4] The complex pathophysiology of CKD and associated bone and mineral disorders involve a number of feedback loops between the kidneys, parathyroid glands, bones, intestine, and vasculature. These alterations occur early during the course of CKD before the onset of clinically detectable abnormalities in bone and vascular system. With progressive loss of kidney function, urinary phosphate excretion and serum phosphate concentration are initially maintained by reducing the proximal tubular reabsorption of filtered phosphate in the remaining functioning nephrons, an effect mediated mainly by compensatory increases in both circulating PTH and FGF23 concentrations. In addition to its phosphaturic effect, FGF23 also suppresses renal synthesis of 1,25(OH)_2_D by inhibiting expression of the enzyme 1-alpha-hydroxylase (CYP27B1), while stimulating the catabolic enzyme 24-hydroxylase (CYP24A1). Hyperphosphatemia, however, is eventually observed in patients with advanced stages of CKD, when the estimated glomerular filtration rate (eGFR) declines below 30 mL/min/1.73 m^2^.[5] It is not known whether other circulating phosphaturic factors (“phosphatonins”) increase as a result of reduced renal clearance or rise to compensate for phosphate retention in patients with CKD.

Fibroblast growth factor (FGF7), also known as heparin-binding growth factor 7 or keratinocyte growth factor, is a 28-kDa protein with phosphatonin-like activity,[6] which is normally expressed in keratinocytes and various epithelial cells.[7, 8] FGF7 prevents epithelial cell injury from reactive oxygen derivatives and promotes wound healing. Deletion of FGF7 in mice alters kidney morphogenesis, resulting in abnormally small ureteric buds and fewer nephrons.[9] FGF7 has been shown to be overexpressed in some patients with tumor-induced (oncogenic) osteomalacia, causing renal phosphate wasting and refractory hypophosphatemia.[10, 11] Moreover, a recent study revealed that serum FGF7 concentrations in pediatric patients with hypophosphatasia and hyperphosphatemia were significantly lower than in control group (27.0 ± 7.7 versus 38.4 ± 3.0 pg/mL, *P* < 0.0001), and intravenous administration of recombinant FGF7 caused phosphaturia in rats, suggesting that FGF7 insufficiency could contribute to hyperphosphatemia in pediatric hypophosphatasia.[12].

There is no data available regarding serum FGF7 concentrations in patients with CKD. We hypothesized that the phosphatonin FGF7 might play a role in compensating for the elevation of serum phosphate concentrations in patients with CKD. Furthermore, FGF7 may be elevated in response to diminished renal clearance. Therefore, we performed a cross-sectional study to determine the relationship between serum intact FGF7 (iFGF7) concentration, eGFR, and associated factors in patients with a broad range of kidney function.

## Methods

This cross-sectional study was performed in accordance with the principles of the Declaration of Helsinki and was approved by the Mayo Clinic Institutional Review Board.As we collected residual blood drawn for routine clinical laboratory tests and the study involved no more than minimal risk, the need for obtaining informed consent was waived. Non-identified information was used to protect patient data confidentiality. Serum samples were collected from 85 non-dialysis patients aged 18 years or older with varying eGFR at Mayo Clinic in Rochester, Minnesota, from September through November 2019. Patients were excluded when they had a known history of phosphate wasting disorders (e.g., tumor-induced osteomalacia, X-linked hypophosphatemic rickets, autosomal dominant hypophosphatemic rickets), hypophosphatasia, primary hyperparathyroidism, were being treated with activated forms of vitamin D (calcitriol or paricalcitol) or calcimimetics (cinacalcet or etelcalcetide), or were currently receiving renal replacement therapy (hemodialysis, peritoneal dialysis, or kidney transplant). The Chronic Kidney Disease Epidemiology Collaboration (CKD-EPI) creatinine equation was used to classify CKD severity (CKD1: eGFR ≥ 90; CKD2: eGFR 60–89; CKD3a: eGFR 45–59; CKD3b: eGFR 30–44; CKD4: eGFR 15–29; and CKD5: eGFR < 15 mL/min/1.73 m^2^). Patient serum samples were stored at − 80^o^C until analysis.

Laboratory parameters, including hemoglobin, fasting plasma glucose, blood urea nitrogen, serum creatinine, albumin, electrolytes, calcium, phosphate, and total alkaline phosphatase concentration, were measured using standard automated assays in the Central Clinical Laboratory at Mayo Clinic. Serum PTH concentrations were measured using a second-generation iPTH immunoassay and a Roche Cobas e411 analyzer (Roche Diagnostics, Mannheim, Germany). Serum 25(OH)D and 1,25(OH)_2_D concentrations were measured using liquid chromatography-tandem mass spectrometry (LC-MS/MS). Serum iFGF7 and iFGF23 concentrations were measured using the recombinant human iFGF7 enzyme-linked immunosorbent assay (R&D Systems, MN, USA) and the human iFGF23 enzyme-linked immunosorbent assay (Eagle Biosciences, NH, USA), respectively. The inter- and intra-assay coefficients of variation (CV) were 6.0 and 3.3 % for the iFGF7 immunoassay, and less than 5 % for the iFGF23 immunoassay, respectively. The lower limits of detection for iFGF7 and iFGF23 immunoassays were 15 and 10 pg/mL, respectively.

### Statistical analysis

Statistical analysis included computing (i) the frequency counts and percentages for the categorical variables and (ii) the means ± standard deviations (SD) or medians with interquartile ranges (IQ25 and IQ75) for the continuous variables. Continuous variables were tested for normality using a Shapiro-Wilk test. For non-normally distributed continuous variables, medians with interquartile ranges (IQ25 and IQ75) were presented. We examined interrelationships of measured serum analytes using nonlinear regression analysis. A segmented regression model was used to detect thresholds at which a statistically significant change was observed in the slope of serum analyte concentrations in relation to eGFR.[13] Univariate regression analyses were performed to determine the associations between serum iFGF7 concentrations and potential factors: age, sex, body mass index (BMI), eGFR, 24-hour urine protein, albumin-corrected serum calcium, serum phosphate, PTH, FGF23, and 1,25(OH)_2_D concentrations. Only factors that had univariate associations of *P* values < 0.25 were further considered in subsequent multivariate models.[14] Lack of collinearity was confirmed by testing variance inflation factors. All tests were two-sided and *P* values < 0.05 were considered statistically significant. All statistical analysis was performed using STATA, version 14.0 (TX, USA).

## Results

A cohort of 85 patients with a broad range of eGFR was recruited (Table 1). The mean (± SD) age was 58.2 ± 16.6 years, and 57.6 % of patients were female. The mean (± SD) eGFR was 53.9 ± 31.7 mL/min/1.73 m^2^, the median (IQR) 24-hour urine protein was 347 (198-1,485) mg/24 h, and 28.2 % of patients had received a diagnosis of type 2 diabetes. A total of 17.6 % of patients had low serum 25(OH)D concentrations (< 30 ng/mL).

**Table 1:** Clinical characteristics of the study patients, according to stages of CKD

Characteristics	Estimated GFR (mL/min/1.73 m^2^)				
	≥ **60 (*n*** **= 31)**	**45–59 (*n*** **= 16)**	**30–44 (*n*** **= 11)**	**15–29 (*n*** **= 15)**	**< 15 (*n*** **= 12)**
Age (yr)	51.1 ± 17.2	60.2 ± 15.3	60.4 ± 14.9	63.6 ± 16.5	67.1 ± 12.0
Female sex (no. [%])	21 (67.7)	9 (56.3)	7 (63.6)	8 (53.3)	4 (33.3)
Diabetes (no. [%])	6 (19.4)	5 (31.3)	4 (36.4)	4 (26.7)	5 (41.7)
Calcium use (no. [%])	8 (25.8)	4 (25.0)	2 (18.2)	4 (26.7)	4 (33.3)
Cholecalciferol or ergocalciferol use (no. [%])	13 (41.9)	6 (37.5)	3 (27.3)	4 (26.7)	6 (50)
Body mass index (kg/m^2^)	28.1 ± 6.0	26.7 ± 6.1	27.8 ± 5.7	29.5 ± 6.3	32.2 ± 9.2
Systolic blood pressure (mmHg)	120.9 ± 13.7	127.7 ± 16.8	122.6 ± 21.6	125.7 ± 11.7	135.5 ± 16.0
Hemoglobin (g/dL)	12.6 ± 1.4	11.9 ± 1.7	11.5 ± 1.0	11.0 ± 2.0	10.6 ± 1.2
Fasting plasma glucose (mg/dL)	109 ± 23	113 ± 32	109 ± 20	117 ± 30	124 ± 42
Blood urea nitrogen (mg/dL)	16.4 ± 4.6	21.3 ± 5.4	24.9 ± 7.4	44.5 ± 8.5	54.4 ± 14.4
Estimated GFR (mL/min/1.73 m^2^)	88.1 ± 17.3	51.4 ± 4.8	37.1 ± 3.2	23.7 ± 4.4	12.8 ± 1.5
Serum sodium (mEq/L)	140.4 ± 2.9	140.8 ± 1.7	140.2 ± 2.5	140.5 ± 2.4	141.4 ± 2.8
Serum potassium (mEq/L)	4.2 ± 0.5	4.6 ± 0.4	4.4 ± 0.4	4.5 ± 0.6	4.6 ± 0.3
Serum chloride (mEq/L)	103.1 ± 3.0	103.9 ± 1.7	104.9 ± 4.6	104.3 ± 3.1	103.7 ± 5.8
Serum bicarbonate (mEq/L)	24.5 ± 2.0	24.0 ± 1.4	22.1 ± 2.5	23.7 ± 4.4	21.9 ± 2.9
Corrected serum calcium (mg/dL)^a^	9.5 ± 0.5	9.4 ± 0.4	9.4 ± 0.5	9.4 ± 0.4	9.3 ± 0.5
Serum phosphate (mg/dL)	3.3 ± 0.4	3.3 ± 0.7	3.5 ± 0.6	4.0 ± 0.6	4.7 ± 0.3
Serum alkaline phosphatase (U/L)^b^	85 (61–100)	58 (52–91)	64 (53–80)	104 (70–112)	92 (76–116)
24-hour urine protein (mg/day)^b^	227 (147–377)	185 (135–410)	287 (252–502)	611 (347-2,327)	2561 (1554–4208)
Serum parathyroid hormone (pg/mL)^b^	63 (47–85)	66 (45–83)	72 (46–93)	115 (92–168)	246 (143–393)
Serum 25(OH)D (ng/mL)	38.8 ± 11.9	49.9 ± 12.3	37.1 ± 11.9	42.2 ± 13.0	41.8 ± 15.7
Serum 1,25(OH)_2_D (pg/mL)	41.5 ± 12.7	33.5 ± 12.5	29.8 ± 11.6	21.7 ± 8.4	15.6 ± 1.8
Serum intact FGF7 (pg/mL)^b^	46.1 (39.2–56.9)	43.1 (39.0-51.5)	47.3 (38.3–66.5)	47.7 (37.7–55.8)	49.6 (42.5–65.6)
Serum intact FGF23 (pg/mL)^b^	41.9 (33.0-52.7)	56.4 (46.9–60.2)	62.9 (50.9–79.3)	117.5 (88.4-156.6)	415.9 (278.3-500.9)

For continuous variables, mean ± SD; for categorical variables, *n* (%); unless otherwise specified

^a^Corrected serum calcium (mg/dL) = measured total calcium (mg/dL) + 0.8(4.0 – serum albumin (g/dL))

^b^Shown as median (interquartile range, 25–75 %)

### Changes in biomarkers of Mineral Metabolism across CKD stages

**Fig. 1:**
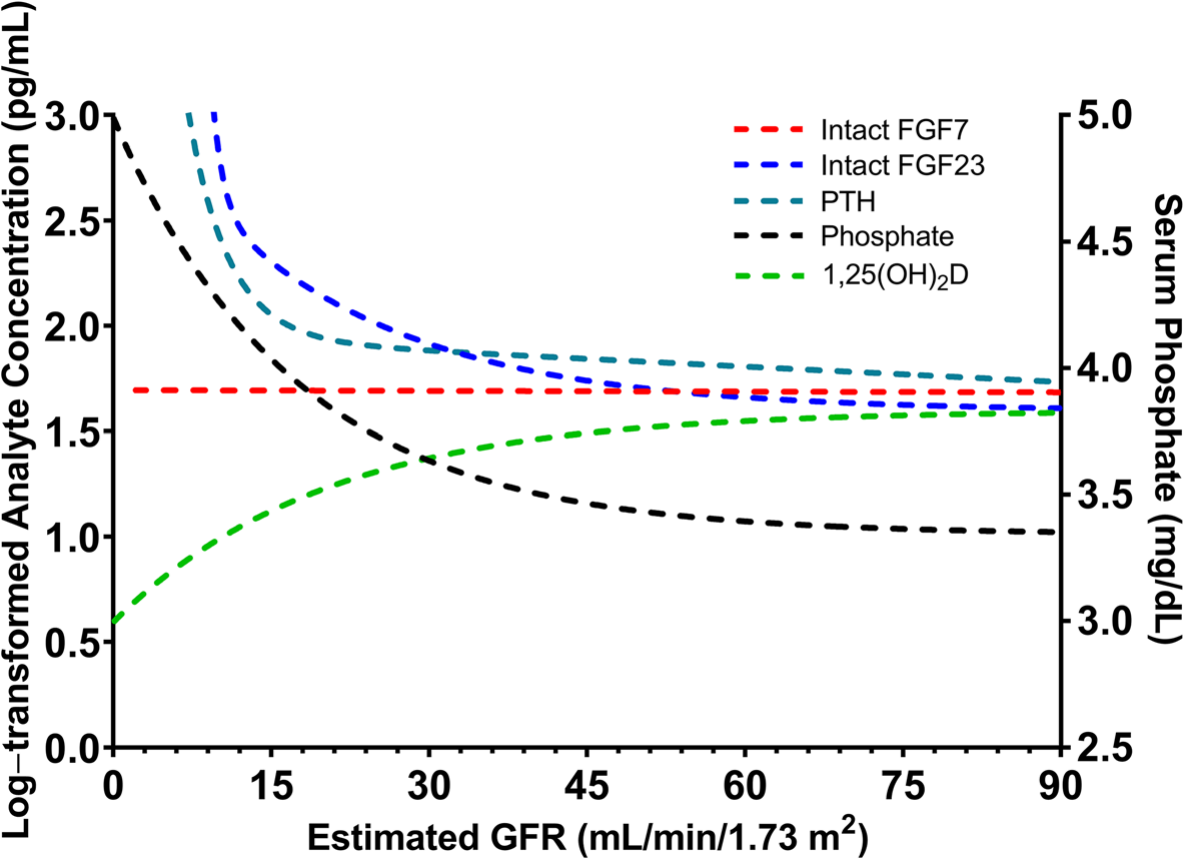
Correlations between estimated GFR and serum concentrations of mineral metabolism biomarkers: iFGF7 (*R*^2^ = 0.10; *P* = 0.28), iFGF23 (*R*^2^ = 0.44; *P* < 0.01), PTH (*R*^2^ = 0.40; *P* < 0.01), phosphate (*R*^2^ = 0.32; *P* < 0.01), and 1,25(OH)_2_D (*R*^2^ = 0.35; *P* < 0.01)

**Fig. 2:**
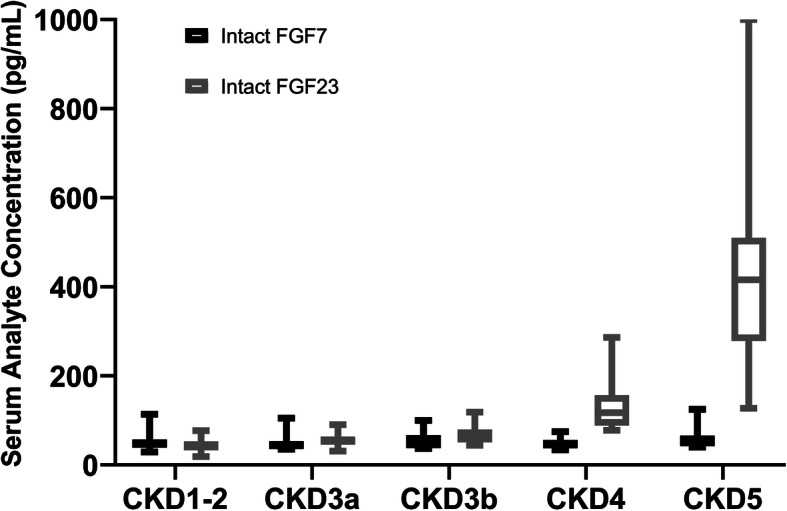
Serum concentrations of iFGF7 and iFGF23 in each CKD stage. Boxes represent the interquartile range with the upper and lower edges representing the 75th and 25th percentiles, respectively. The central horizontal lines represent the median serum concentrations. Only CKD4-5 demonstrate a statistically significant increase in serum iFGF23 concentrations relative to CKD1-3 (*P* < 0.01)

As eGFR declined, we observed a progressive increase of serum iFGF23, PTH, and phosphate concentrations and a progressive reduction in serum 1,25(OH)_2_D concentrations. Interestingly, serum iFGF7 concentrations remained relatively unchanged (Figs. 1 and 2). There was no significant association between kidney function and albumin-corrected serum calcium, total alkaline phosphatase, or 25(OH)D concentrations.

**Fig. 3:**
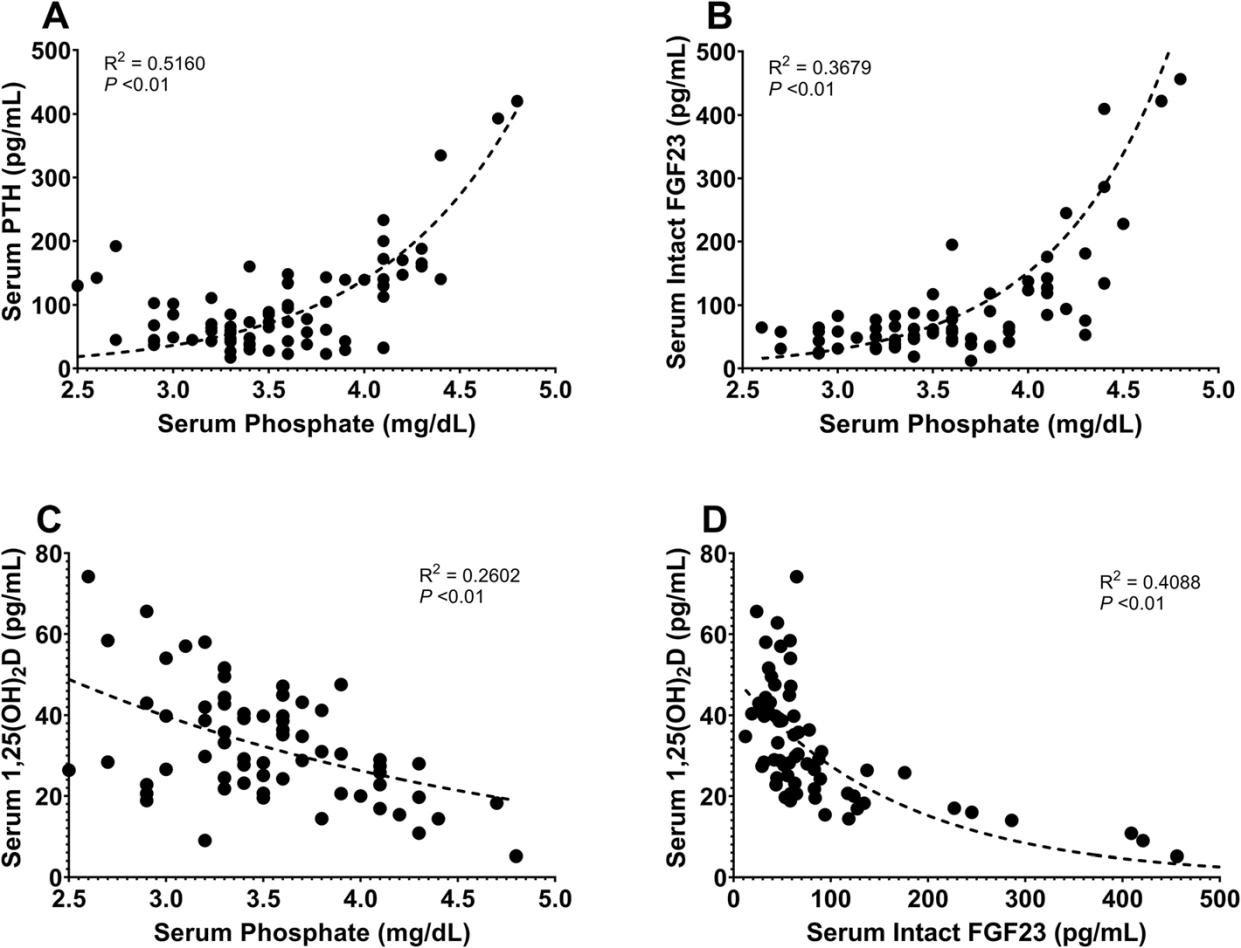
Correlations of serum phosphate concentrations with serum PTH, iFGF23, and 1,25(OH)_2_D concentrations (**a**-**c**), and correlations between serum iFGF23 and 1,25(OH)_2_D concentrations (**d**)

Significant increases in serum concentrations of iFGF23, PTH, and phosphate were observed at eGFRs of < 33 (95 % CI, 26.40-40.05), < 29 (95 % CI, 22.51–35.36) and < 22 mL/min/1.73 m^2^ (95 % CI, 19.25–25.51), respectively. Moreover, a significant decrease in serum 1,25(OH)_2_D concentrations was observed at an eGFR of < 52 mL/min/1.73 m^2^ (95 % CI, 42.57–61.43). We observed a positive correlation between serum phosphate and PTH concentrations (*R*^2^ = 0.5160; *P* < 0.01; Fig. 3 a) and a positive correlation between serum phosphate and iFGF23 concentrations (*R*^2^ = 0.3679; *P* < 0.01; Fig. 3b). Additionally, we observed a negative correlation between serum phosphate and 1,25(OH)_2_D concentrations (*R*^2^ = 0.2602; *P* < 0.01; Fig. 3 c) and a negative correlation between serum iFGF23 and 1,25(OH)_2_D concentrations (*R*^2^ = 0.4088; *P* < 0.01; Fig. 3d).

### Serum concentration of iFGF7 in patients with CKD

The median (IQR) serum iFGF7 concentration was 46.2 (39.4–56.7) pg/mL and did not significantly differ across the stages of CKD. For eGFRs of ≥ 60, 45–59, 30–44, 15–29, and < 15 mL/min/1.73 m^2^, median (IQ25-75) serum iFGF7 concentrations were 46.1 (39.2–56.9), 43.1 (39.0-51.5), 47.3 (38.3–66.5), 47.7 (37.7–55.8), and 49.6 (42.5–65.6) pg/mL, respectively (*P* = 0.62). There was no significant correlation between serum iFGF7 concentrations and serum phosphate, PTH, iFGF23, 25(OH)D, or 1,25(OH)_2_D concentrations. We stratified patients by quartiles of serum iFGF7 concentration: 22 subjects were in the 1st quartile (serum iFGF7 < 39.4 pg/mL), 21 were in the 2nd quartile (39.4 ≤ serum iFGF7 < 46.4 pg/mL), 21 were in the 3rd quartile (46.4 ≤ serum iFGF7 < 56.7 pg/mL), and 21 were in the 4th quartile (serum iFGF7 ≥ 56.7 pg/mL). Multiple logistic regression analysis that adjusted for sex, age, BMI, eGFR, serum phosphate, and 1,25(OH)_2_D concentrations revealed that higher BMI was independently associated with the highest quartile of serum iFGF7 concentration (OR per 5 kg/m^2^ increase in BMI, 1.20; 95 % CI, 1.12–1.55; Table 2).

**Table 2:** Factors associated with highest intact FGF7 quartile by multivariate analysis

Variables	Adjusted Odds Ratio	*P* Value	95 % Confidence Interval
Male sex	0.47	0.22	0.14–1.56
Age (per 5 year increase)	0.86	0.13	0.73–1.07
Body mass index (per 5 kg/m^2^ increase)	1.20	0.01	1.12–1.55
Estimated GFR (per 5 mL/min/1.73 m^2^ increase)	0.89	0.06	0.79–1.09
Serum phosphate (per 1 mg/dL increase)	0.36	0.06	0.17–1.04
Serum 1,25(OH)_2_D (per 1 pg/mL increase)	1.03	0.23	0.98–1.06

Quartile cutpoints for serum intact FGF7 concentration: 39.4, 46.4, 56.7 pg/mL

## Discussion

In this cross-sectional study of non-dialysis patients with varying degrees of kidney dysfunction, we show, for the first time, that serum concentrations of the phosphatonin iFGF7 are not significantly altered across the stages of CKD. Based on multiple logistic regression analyses, we found that elevated serum iFGF7 concentrations are associated with higher BMI. This research confirms previous findings that a decrease in serum 1,25(OH)_2_D concentrations and an increase in serum iFGF23 concentrations occur in early stages of CKD, whereas increases in serum PTH and phosphate concentrations are observed at later stages of CKD.

Fibroblast growth factor (FGF7), also known as heparin-binding growth factor 7 or keratinocyte growth factor, is a 28-kDa protein of the FGF family which is highly expressed in various epithelial cells including keratinocytes. FGF7 acts as an autocrine or paracrine growth factor by activating the FGFR2b receptor on epithelial cell membranes.[15, 16] FGF7 plays a role in morphogenesis (e.g., kidney development), angiogenesis, tissue repair, and tumorigenesis.[9, 17–19] FGF7 expression is also upregulated in kidney tissues from patients with autosomal dominant polycystic kidney disease.[20] Several reports demonstrate the phosphatonin-like activity of FGF7 both *in vitro* and *in vivo*. A previous study demonstrated that FGF7 is overexpressed in some patients with mesenchymal tumors associated with osteomalacia, renal phosphate wasting, and hypophosphatemia.[6] The investigators found that FGF7 in tumor cell cultures inhibited *in vitro* sodium-dependent phosphate transport in opossum kidney proximal tubule cells, and that this inhibitory activity of tumor-derived cultures was abrogated by coincubation with neutralizing anti-FGF7 antibodies. Additionally, Bansal et al. described a patient with tumor-induced osteomalacia and elevations in serum concentrations of both FGF7 and FGF23.[11] Interestingly, a recent study showed that low serum FGF7 concentrations were observed in pediatric patients with hypophosphatasia and hyperphosphatemia.[12] Moreover, the intravenous FGF7 administration increased the fractional excretion of phosphate *in vivo* in rats, suggesting that hyperphosphatemia in pediatric hypophosphatasia might be partly attributable to FGF7 insufficiency.

To our knowledge, the circulating concentration of the phosphatonin FGF7 in patients with CKD has not been previously examined, and it is unknown whether FGF7 increases urinary phosphate excretion and facilitates normalization of serum phosphate concentration in early CKD. Our study failed to find evidence for compensatory increases in serum concentrations of FGF7 across CKD stages, suggesting that FGF7 may not play an important role in the pathophysiology of CKD-MBD in non-dialysis patients. Although a renal clearance study in human demonstrated the single-pass renal extraction of FGF23 (17.1 %±19.5 %) and PTH (44.2 %±10.3 %),[21] the data on renal FGF7 clearance are lacking. We postulate that FGF7 clearance may not be affected by kidney function. Furthermore, some uremic toxins may suppress phosphate-induced FGF7 production in patients with advanced CKD, thereby blunting compensatory increases in serum FGF7 concentrations. Alternatively, phosphate-sensing receptor might be absent in FGF7-producing cells. Further studies are needed to determine which one of proposed possibilities contributes to a lack of change in serum FGF7 concentrations in CKD. This study has shown that circulating iFGF7 concentration was independently associated with increased BMI. The adipose tissue may contribute to the generation of FGF7 or, alternatively, FGF7 could affect adipocyte metabolism. The exact mechanism underlying the correlation between serum FGF7 concentration and body fat mass needs further study.

Consistent with previous studies, we found that low serum concentrations of 1,25(OH)_2_D occur earlier in the course of the eGFR decline than do elevations in serum PTH concentrations.[22, 23] A reduction in renal mass, and accumulation of various factors such as FGF23,[24–26] phosphate,[27, 28] PTH fragments,[29] and unmeasured uremic toxins could suppress the activity of renal 1-alpha-hydroxylase, thereby contributing to a reduction in serum 1,25(OH)_2_D concentrations. Our results did not show significant relationships of serum 25(OH)D and 1,25(OH)_2_D concentrations or kidney function, which are consistent with previous reports.[22, 25, 30, 31] These findings imply that the activity of renal 1-alpha-hydroxylase, rather than the abundance of circulating 25(OH)D, is the primary determinant of serum 1,25(OH)_2_D concentration. Low serum concentrations of 1,25(OH)_2_D augment PTH secretion by indirect and direct mechanisms. Indirect effects on PTH secretion occur through diminished intestinal absorption of calcium resulting in hypocalcemia. In addition, low 1,25(OH)_2_D concentrations reduce the expression of the vitamin D receptor in parathyroid glands[32] and result in lower 1,25(OH)_2_D-mediated suppression of PTH secretion.[33] Notably, the low prevalence of hyperphosphatemia in our cohort might be responsible for the lower PTH increase noted at reduced eGFR level in comparison to previous studies.[5, 34] The inter-assay variability in PTH measurements could also account for the difference in the eGFR cutpoint at which serum PTH concentrations increase.[35] This study confirms previous findings that a compensatory increase in circulating FGF23 concentrations commences before the occurrence of hyperphosphatemia.[5, 25] Nevertheless, in previous studies in which FGF23 increases occurred at relatively high eGFR levels, serum FGF23 concentration was measured using a C-terminal FGF23 immunoassay, which recognizes both iFGF23 and C-terminal FGF23 fragments.[5, 25, 36] We speculate that these findings are due to the impaired clearance of C-terminal FGF23 fragment relative to the clearance of iFGF23 in patients with CKD.[37–39].

The main strength of the current study is the comprehensive measurement of mineral metabolism biomarkers, including full-length biologically active forms of FGF7 and FGF23 as well as 1,25(OH)_2_D, among patients with varying levels of eGFR. Our study also has some limitations. The biochemical data on other circulating phosphatonins, including matrix extracellular phosphoglycoprotein and secreted frizzled-related protein 4 (sFRP4), are lacking. Nonetheless, in a small study of patients with CKD, serum sFRP4 concentrations did not significantly change with creatinine clearance or serum phosphate concentrations.[40] In addition, our cohort comprised only non-dialysis patients; thus, these data may not be generalizable to patients receiving dialysis or to kidney transplant recipients. Moreover, cross-sectional analyses cannot determine causality.

## Conclusions

The normalization of serum phosphate concentrations in early stages of CKD may be mainly mediated by compensatory decreases in circulating concentrations of 1,25(OH)_2_D and increases in circulating concentrations of iFGF23 and PTH, while the phosphatonin iFGF7 may not play a crucial role in the compensatory response to phosphate retention in CKD-MBD. The longitudinal changes of mineral metabolism biomarkers and other circulating phosphatonins during the course of CKD need to be further explored.

## Data Availability

The dataset generated and/or analyzed during the current study is available from the corresponding author on reasonable request.

## Abbreviations

1,25(OH)_2_D: 1,25-hydroxyvitamin D
25(OH)D: 25-hydroxyvitamin D
BMI: Body mass index
CKD: Chronic kidney disease
eGFR: Estimated glomerular filtration rate
FGF: Fibroblast growth factor
MBD: Mineral and bone disorder
PTH: Parathyroid hormone
sFRP4: Secreted frizzled-related protein 4

## References

[1] Covic A, Vervloet M, Massy ZA, Torres PU, Goldsmith D, Brandenburg V (2018). Bone and mineral disorders in chronic kidney disease: implications for cardiovascular health and ageing in the general population. Lancet Diabetes Endocrinol..

[2] Bover J, Ureña-Torres P, Mateu S, DaSilva I, Gràcia S, Sánchez-Baya M (2020). Evidence in chronic kidney disease-mineral and bone disorder guidelines: is it time to treat or time to wait?. Clin Kidney J.

[3] Ketteler M, Block GA, Evenepoel P, Fukagawa M, Herzog CA, McCann L (2017). Executive summary of the 2017 KDIGO Chronic Kidney Disease-Mineral and Bone Disorder (CKD-MBD) Guideline Update: what’s changed and why it matters. Kidney Int.

[4] Go AS, Chertow GM, Fan D, McCulloch CE, Hsu C (2004). Chronic kidney disease and the risks of death, cardiovascular events, and hospitalization. N Engl J Med.

[5] Isakova T, Wahl P, Vargas GS, Gutiérrez OM, Scialla J, Xie H (2011). Fibroblast growth factor 23 is elevated before parathyroid hormone and phosphate in chronic kidney disease. Kidney Int.

[6] Carpenter TO, Ellis BK, Insogna KL, Philbrick WM, Sterpka J, Shimkets R (2005). Fibroblast growth factor 7: an inhibitor of phosphate transport derived from oncogenic osteomalacia-causing tumors. J Clin Endocrinol Metab.

[7] Beer HD, Gassmann MG, Munz B, Steiling H, Engelhardt F, Bleuel K, et al. Expression and function of keratinocyte growth factor and activin in skin morphogenesis and cutaneous wound repair. J Investig Dermatol Symp Proc. 2000;5:34–9. 10.1046/j.1087-0024.2000.00009.x 11147673

[8] Danilenko DM (1999). Preclinical and early clinical development of keratinocyte growth factor, an epithelial-specific tissue growth factor. Toxicol Pathol.

[9] Qiao J, Uzzo R, Obara-Ishihara T, Degenstein L, Fuchs E, Herzlinger D (1999). FGF-7 modulates ureteric bud growth and nephron number in the developing kidney. Dev Camb Engl.

[10] Marcucci G, Masi L, Ferrarì S, Haffner D, Javaid MK, Kamenický P (2018). Phosphate wasting disorders in adults. Osteoporos Int.

[11] Bansal S, Khazim K, Suri R, Martin D, Werner S, Fanti P (2016). Tumor induced osteomalacia: associated with elevated circulating levels of fibroblast growth factor-7 in addition to fibroblast growth factor-23. Clin Nephrol.

[12] Whyte MP, Zhang F, Wenkert D, Mumm S, Berndt TJ, Kumar R (2020). Hyperphosphatemia with low FGF7 and normal FGF23 and sFRP4 levels in the circulation characterizes pediatric hypophosphatasia. Bone.

[13] Pastor R, Guallar E (1998). Use of two-segmented logistic regression to estimate change-points in epidemiologic studies. Am J Epidemiol.

[14] Mickey RM, Greenland S (1989). The impact of confounder selection criteria on effect estimation. Am J Epidemiol.

[15] Finch PW, Rubin JS (2004). Keratinocyte growth factor/fibroblast growth factor 7, a homeostatic factor with therapeutic potential for epithelial protection and repair. Adv Cancer Res.

[16] Ornitz DM, Marie PJ (2002). FGF signaling pathways in endochondral and intramembranous bone development and human genetic disease. Genes Dev.

[17] Finch PW, Rubin JS (2006). Keratinocyte growth factor expression and activity in cancer: implications for use in patients with solid tumors. J Natl Cancer Inst.

[18] Werner S (1998). Keratinocyte growth factor: a unique player in epithelial repair processes. Cytokine Growth Factor Rev.

[19] Werner S, Krieg T, Smola H (2007). Keratinocyte-fibroblast interactions in wound healing. J Invest Dermatol.

[20] Mei C, Mao Z, Shen X, Wang W, Dai B, Tang B (2005). Role of keratinocyte growth factor in the pathogenesis of autosomal dominant polycystic kidney disease. Nephrol Dial Transplant.

[21] van Ballegooijen AJ, Rhee EP, Elmariah S, de Boer IH, Kestenbaum B (2016). Renal Clearance of Mineral Metabolism Biomarkers. J Am Soc Nephrol.

[22] Levin A, Bakris GL, Molitch M, Smulders M, Tian J, Williams LA (2007). Prevalence of abnormal serum vitamin D, PTH, calcium, and phosphorus in patients with chronic kidney disease: results of the study to evaluate early kidney disease. Kidney Int..

[23] Koenig KG, Lindberg JS, Zerwekh JE, Padalino PK, Cushner HM, Copley JB (1992). Free and total 1,25-dihydroxyvitamin D levels in subjects with renal disease. Kidney Int.

[24] Shimada T, Hasegawa H, Yamazaki Y, Muto T, Hino R, Takeuchi Y (2004). FGF-23 is a potent regulator of vitamin D metabolism and phosphate homeostasis. J Bone Miner Res.

[25] Gutierrez O, Isakova T, Rhee E, Shah A, Holmes J, Collerone G (2005). Fibroblast growth factor-23 mitigates hyperphosphatemia but accentuates calcitriol deficiency in chronic kidney disease. J Am Soc Nephrol.

[26] Saito H, Kusano K, Kinosaki M, Ito H, Hirata M, Segawa H (2003). Human fibroblast growth factor-23 mutants suppress Na+-dependent phosphate co-transport activity and 1alpha,25-dihydroxyvitamin D3 production. J Biol Chem.

[27] Hughes MR, Brumbaugh PF, Hussler MR, Wergedal JE, Baylink DJ (1975). Regulation of serum 1alpha,25-dihydroxyvitamin D3 by calcium and phosphate in the rat. Science.

[28] Tanaka Y, Deluca HF (1973). The control of 25-hydroxyvitamin D metabolism by inorganic phosphorus. Arch Biochem Biophys.

[29] Usatii M, Rousseau L, Demers C, Petit J-L, Brossard J-H, Gascon-Barré M (2007). Parathyroid hormone fragments inhibit active hormone and hypocalcemia-induced 1,25(OH)2D synthesis. Kidney Int.

[30] Ishimura E, Nishizawa Y, Inaba M, Matsumoto N, Emoto M, Kawagishi T (1999). Serum levels of 1,25-dihydroxyvitamin D, 24,25-dihydroxyvitamin D, and 25-hydroxyvitamin D in nondialyzed patients with chronic renal failure. Kidney Int.

[31] LaClair RE, Hellman RN, Karp SL, Kraus M, Ofner S, Li Q (2005). Prevalence of calcidiol deficiency in CKD: a cross-sectional study across latitudes in the United States. Am J Kidney Dis.

[32] Denda M, Finch J, Brown AJ, Nishii Y, Kubodera N, Slatopolsky E (1996). 1,25-dihydroxyvitamin D3 and 22-oxacalcitriol prevent the decrease in vitamin D receptor content in the parathyroid glands of uremic rats. Kidney Int.

[33] Brumbaugh PF, Hughes MR, Haussler MR (1975). Cytoplasmic and nuclear binding components for 1alpha25-dihydroxyvitamin D3 in chick parathyroid glands. Proc Natl Acad Sci U S A.

[34] Muntner P, Jones TM, Hyre AD, Melamed ML, Alper A, Raggi P (2009). Association of serum intact parathyroid hormone with lower estimated glomerular filtration rate. Clin J Am Soc Nephrol.

[35] Souberbielle J-C, Boutten A, Carlier M-C, Chevenne D, Coumaros G, Lawson-Body E (2006). Inter-method variability in PTH measurement: implication for the care of CKD patients. Kidney Int.

[36] Isakova T, Cai X, Lee J, Mehta R, Zhang X, Yang W (2020). Longitudinal Evolution of Markers of Mineral Metabolism in Patients With CKD: The Chronic Renal Insufficiency Cohort (CRIC) Study. Am J Kidney Dis.

[37] Shimada T, Urakawa I, Isakova T, Yamazaki Y, Epstein M, Wesseling-Perry K (2010). Circulating fibroblast growth factor 23 in patients with end-stage renal disease treated by peritoneal dialysis is intact and biologically active. J Clin Endocrinol Metab.

[38] Chudek J, Kocełak P, Owczarek A, Bożentowicz-Wikarek M, Mossakowska M, Olszanecka-Glinianowicz M (2014). Fibroblast growth factor 23 (FGF23) and early chronic kidney disease in the elderly. Nephrol Dial Transplant.

[39] Bożentowicz-Wikarek M, Owczarek A, Kocełak P, Olszanecka-Glinianowicz M, Więcek A, Chudek J (2016). C-Terminal to Intact Fibroblast Growth Factor 23 Ratio in Relation to Estimated Glomerular Filtration Rate in Elderly Population. Kidney Blood Press Res.

[40] Pande S, Ritter CS, Rothstein M, Wiesen K, Vassiliadis J, Kumar R (2006). FGF-23 and sFRP-4 in chronic kidney disease and post-renal transplantation. Nephron Physiol.

